# Combined Electrochemical, Raman Analysis and Machine Learning Assessments of the Inhibitive Properties of an 1,3,4-Oxadiazole-2-Thiol Derivative against Carbon Steel Corrosion in HCl Solution

**DOI:** 10.3390/ma15062224

**Published:** 2022-03-17

**Authors:** Simona Varvara, Camelia Berghian-Grosan, Gianina Damian, Maria Popa, Florin Popa

**Affiliations:** 1 Department of Cadastre, Civil and Environmental Engineering, “1 Decembrie 1918” University of Alba Iulia, 15-17 Unirii Street, 510009 Alba Iulia, Romania; gianina.damian@uab.ro (G.D.); mpopa@uab.ro (M.P.); 2 National Institute for Research and Development of Isotopic and Molecular Technologies, 67-103 Donat Street, 400293 Cluj-Napoca, Romania; 3 Materials Science and Engineering Department, Technical University of Cluj-Napoca, 103-105 Muncii Avenue, 400641 Cluj-Napoca, Romania; florin.popa@stm.utcluj.ro

**Keywords:** carbon steel, corrosion, 1,3,4-oxadiazole derivative, electrochemical impedance spectroscopy, polarization curves, SEM-EDX, Raman spectroscopy, machine learning algorithms

## Abstract

The inhibiting properties of 5-(4-pyridyl)-1,3,4-oxadiazole-2-thiol (PyODT) on the corrosion of carbon steel in 1.0 M HCl solution were investigated by potentiodynamic polarization, electrochemical impedance spectroscopy, Raman spectroscopy, and SEM-EDX analysis. An approach based on machine learning algorithms and Raman data was also applied to follow the carbon steel degradation in different experimental conditions. The electrochemical measurements revealed that PyODT behaves as a mixed-type corrosion inhibitor, reaching an efficiency of about 93.1% at a concentration of 5 mM, after 1 h exposure to 1.0 M HCl solution. Due to the molecular adsorption and structural organization of PyODT molecules on the C-steel surface, higher inhibitive effectiveness of about 97% was obtained at 24 h immersion. The surface analysis showed a significantly reduced degradation state of the carbon steel surface in the presence of PyODT due to the inhibitor adsorption revealed by Raman spectroscopy and the presence of N and S atoms in the EDX spectra. The combination of Raman spectroscopy and machine learning algorithms was proved to be a facile and reliable tool for an incipient identification of the corrosion sites on a metallic surface exposed to corrosive environments.

## 1. Introduction

Due to its excellent mechanical properties, low price, and easy fabrication, carbon steel (C-steel) has a broad range of applications in several industrial fields, such as petroleum production and refining, chemical processing, marine, building materials, pipelines, heat exchangers, cooling water systems, metal-processing equipment, etc. [1]. However, C-steel materials are prone to severe degradation in the acid solutions (i.e., HCl, H_2_SO_4_.) that are commonly used for the removal of undesirable rust and scale in various chemical and industrial processes, such as acid pickling, acid cleaning, acid descaling, and oil well acidizing [2]. The acid-induced corrosion phenomenon affects the lifespan of industrial equipment, causing substantial economic losses, environmental problems, and even safety hazards for humans. 

The use of inhibitors is one of the most effective, practical, and economical methods to mitigate corrosion risks [3]. A wide range of inorganic (i.e., nitrites, phosphates, chromates, vanadates, zinc salts, etc.) and organic compounds have been investigated as potential corrosion inhibitors of steel materials in acid media [4,5,6,7].

Although phosphate and chromate-based treatments are among the most inexpensive and efficient for C-steel protection against corrosion, they are not environmentally compliant since phosphorous is a critical contaminant of water bodies, leading to their eutrophication [8]. At the same time, chromium (VI) is highly toxic with carcinogenic effects on humans [9]. Likewise, the vanadate-based coatings do not meet environmental and health restrictions since vanadium compounds were proved as carcinogenic [9].

As compared to the inorganic inhibitors, the organic compounds are very efficient in small concentrations and might provide better film-forming properties [10]. Consequently, most of the acid inhibitors used nowadays for steels protection are organic compounds possessing heteroatoms (N, S, O, or P) with lone electron pairs and/or multiple conjugated bonds with delocalized electrons in their structures, which facilitate the adsorption on the metals surface and the formation of thin protective barrier films [5]. Various classes of organic compounds act as corrosion inhibitors for steels in acidic media [6,11,12,13], including amines, urea, mercaptobenzothiazole, sodium benzoate, imidazolines, surfactants, amides, aldehydes, amino acids, ionic liquids, etc. 

Oxadiazole derivatives represent another class of heterocyclic compounds belonging to the azole family that possesses an abundance of π-electrons and lone electron pairs on the N and O atoms that can interact with the d-orbitals of iron, providing excellent anticorrosive properties. These compounds can be regarded as innoxious inhibitors because of their characteristics of strong chemical activity, as well as low toxicity [14]. 

In the last two decades, 1,3,4-oxadiazole derivatives have been extensively used in pharmaceuticals, dyes, photographic materials, agrochemicals [15]. Likewise, 1,3,4-oxadiazole core presents a broad spectrum of biological activities, including antimicrobial, anti-inflammatory, analgesic, antitumor, anticonvulsant, antidiabetic, fungicidal, herbicidal, antioxidant, anti-HIV, antihypertensive, and anti-obesity [16]. 

In the corrosion field, several 1,3,4-oxadiazole derivatives have been proved as effective corrosion inhibitors for copper-based materials [17,18,19], nickel [20], and steels [21,22,23,24,25,26,27,28,29]. More information on the anticorrosive performances of different 1,3,4-oxadiazole derivatives already studied for steel corrosion in HCl environment is presented in Table S1 from the Supplementary file. Their inhibiting efficiencies are indicated in correlation with the inhibitor nature, inhibitor concentration range, and the adsorption mode. The previous studies showed that 1,3,4-oxadiazole derivatives could adsorb on the steel surface through electrostatic interaction between the charged inhibitor molecules and the charged metal surface (physical adsorption) and/or by the formation of coordinate-type chemical bonds (chemisorption), changing the surface and interface free energies [30]. The adsorbed inhibitor film further mitigates the corrosion process by slowing down the anodic, cathodic, or electrochemical reactions [5]. However, the adsorption of the organic compounds on the metal surface was found to depend on several factors, such as the chemical and electronic structure of the inhibitor molecule (i.e., steric effects, functional groups, electronic density of donor atoms, and π orbital character of donating electrons), the type of the corrosive environment, the nature of the interaction with the d-orbital of iron, temperature, and exposure period [13]. 

Despite the enhanced protection afforded by the corrosion inhibitors, the degradation of the metallic surfaces might occur during a time, especially when the exposure conditions are changed. Therefore, earlier and accurate identification of the incipient corrosion damage of the metallic surface is of particular importance during the monitoring process of the metals’ state. 

Among the experimental methods that can be applied to evaluate the metal’s surface, Raman spectroscopy is a non-destructive technique that can be used both in-situ and air environments [31,32]. However, a large surface analysis is mandatory for a better evaluation of the metals’ area, leading to substantial Raman data collected. Their interpretation represents a great challenge even for experienced researchers.

The use of machine learning algorithms for various techniques, including Raman spectroscopy, has been previously demonstrated as being of great potential for recognizing various features in Raman spectra [33]. Moreover, a methodology based on the combination between Raman spectroscopy and machine learning algorithms has proved its efficiency to discriminate between the Raman profiles of some corroded and inhibitor-protected copper samples in 3.5% NaCl solution [34].

The present study was undertaken to investigate for the first time the potential of 5-(4-pyridyl)-1,3,4-oxadiazole-2-thiol (PyODT) as an effective inhibitor for C-steel corrosion in 1.0 M HCl solution and to assess the degradation state of the metal by combining the Raman spectroscopy and machine learning algorithms. The anticorrosive properties of PyODT were evaluated by electrochemical impedance spectroscopy, potentiodynamic polarization, Raman spectroscopy, scanning electron microscopy, and energy-dispersive X-ray spectrometry analyses. Furthermore, machine learning algorithms applied to the Raman data acquired by analyzing the C-steel surface after different exposure times to the blank and inhibitor-containing solutions were also used to evaluate the metal degradation state and validate the approach as a facile tool for the early identification of the surface corrosion degree. No attempts to apply this approach on such complex systems, as the metallic alloys exposed to a highly corrosive environment have been reported. Due to the easy accessibility of the handheld or mobile Raman spectrometers that have significantly developed in the last decades, this non-destructive technique could be used in combination with the artificial intelligence methods for rapid and incipient identification of the corrosion sites on the metallic surface.

## 2. Materials and Methods

### 2.1. Chemicals and Materials

**Figure 1:**
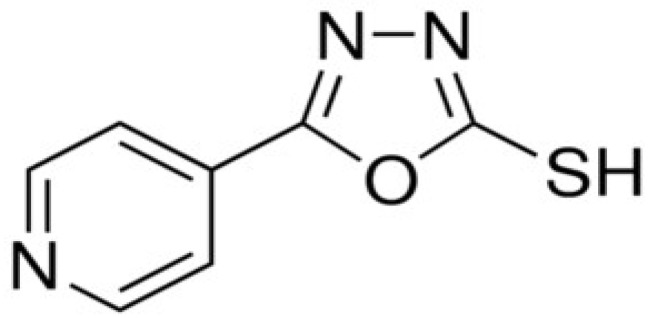
Molecular structure of 5-(4-pyridyl)-1,3,4-oxadiazole-2-thiol.

The corrosive solution (1.0 M HCl) was prepared by dilution of the analytical grade, HCl 37% (Merck, Darmstadt, Germany), with distilled water. The investigated organic compound was 5-(4-pyridyl)-1,3,4-oxadiazole-2-thiol (PyODT) purchased from Alfa Aesar (97%, Lancashire, UK); its molecular structure is presented in Figure 1. 

The inhibitor-containing electrolytes were prepared by dissolving appropriate weighted amounts of PyODT in a mixture of 1.0 M HCl solution and ethanol (90:10, *v*/*v*). The PyODT concentration in the test solution was in the range of 0.1 to 10 mM.

The corrosion tests were conducted on a carbon steel (C-steel) electrode with the following elemental composition (wt.): Mn (0.6%), C (0.32%), Si (0.04%), S (0.029%), P (0.01%) and balance Fe. The metallic cylinder specimen was embedded in epoxy resin (Buhler, Epoxycure, Esslingen am Neckar, Germany), leaving an exposed surface area of 0.5 cm^2^. Before immersion in the corrosive solution, the C-steel surface was prepared by grinding with a successive grade of silicon carbide papers (from 1200 up to 4000 grit) and then polished with 0.3 μm alumina slurry to obtain a mirror-like surface. Finally, the C-steel electrode was rinsed thoroughly with distilled water, dried at room temperature, and immediately introduced in a glass cell containing 100 mL of electrolyte.

### 2.2. Electrochemical Measurements

All electrochemical measurements were performed in a standard three-electrode cell configuration, using a Gill AC potentiostat/galvanostat (ACM Instruments, Cumbria, UK). The C-steel electrode served as a working electrode, while a platinum sheet and an Ag/AgCl/KClsat were used as auxiliary and reference electrodes. Before any electrochemical measurement, the C-steel electrode was left unpolarized at room temperature (298 K) in the electrolyte for 1 h to achieve a steady-state condition. 

Electrochemical impedance spectroscopy measurements (EIS) were performed under the open circuit potential (OCP) condition by applying a sinusoidal frequency perturbation from 10 kHz to 100 mHz with an *ac* voltage amplitude of ±10 mV. The obtained experimental impedance data were modeled using ZSimpWin 3.21 software (Ametek, Berwyn, PA, USA). 

Polarization curves were recorded at a constant sweep rate of 10 mVmin^−1^ in a potential range of ±250 mV vs. OCP, starting from the cathodic to the anodic direction.

### 2.3. SEM-EDX Analysis

For the surface analysis, C-steel specimens were immersed in 1.0 M HCl solution without and with the optimum concentration of PyODT at 298 K. After 1 h and 12 h of exposure, the metallic samples were carefully removed from the electrolyte, gently washed with distilled water, dried at room temperature, and characterized without any further treatment by scanning electron microscopy (SEM). SEM measurements were carried out using a JSM 5600 LV scanning electron microscope (JEOL, Akishima, Tokyo, Japan), operated at an accelerating voltage of 15 kV. The elemental analysis of the corrosion products formed on the C-steel surface was performed by the energy-dispersive X-ray spectroscopy (EDX) using an Oxford Instruments spectrometer (UltimMAx65 (High Wycombe, UK).

### 2.4. Raman Spectroscopy

For the Raman investigations, C-steel electrodes were immersed in 1.0 M HCl solution without or with the optimum concentration of PyODT at room temperature. The corrosive effect of 1.0 M HCl solution on the C-steel surface has been investigated after different exposure times, i.e., 1 h and 6 h in the blank solution and 1 h and 12 h in the presence of 5 mM PyODT. Due to the highly corrosive effect of the uninhibited electrolyte on the metallic surface, a lower exposure period (6 h) has been chosen for the Raman study. Further, the metallic samples were carefully removed from the electrolyte, gently washed with distilled water, dried at room temperature, and characterized by Raman spectroscopy. Raman analyses were performed using a JASCO NRS-3300 (Jasco, Tokyo, Japan) equipped with a CCD detector (−69 °C) and a 785 nm diode laser excitation line. The measurements were achieved using a 100× magnification objective (UMPLFL Olympus, Shinjuku, Tokyo, Japan) and a grid of 600 l/mm, while the 521 cm^−1^ Si peak was used for the calibration procedure. Except for the solid PyODT and C-steel electrode surface, whose structures and homogeneity are free from uncertainty, the other samples were investigated in several points, at least 15, in order to obtain a clear picture of the transformations that occurred at the electrode surface after various treatments. Depending on the electrode surface nature, the spectra were collected using an exposure time of 60 s and 3 or 4 accumulations (for C-steel/ PyODT electrodes), while 10 s and 6 or 12 spectral accumulations were involved for the C-steel/HCl electrodes surfaces. JASCO Spectra Manager (2.07.00, 2002–2008), tools (Jasco, Tokyo, Japan) were employed for spectra analysis and the frequency range selection; for all samples, the spectral domain between 150 and 1820 cm^−1^ has been chosen for investigation. Then, each spectrum was subjected to baseline subtraction, and [0, 1] normalization pre-treatments were performed in OriginPro 2017, b 9.4.0.220 (OriginLab, Northampton, MA, USA).

### 2.5. Machine Learning Studies

For data analysis by the machine learning (ML) algorithms, the normalized Raman data were split into training and testing groups. For the training dataset preparation, 6 classes of interest were chosen: untreated C-steel (CS), C-steel after 1 h and 6 h of immersion in 1.0 M HCl (CS-HCl-1h and CS-HCl-6h), C-steel after 1 h and 12 h of immersion in 1.0 M HCl solution containing 5 mM PyODT (CS-PyODT-1h and CS-PyODT-12h) and the solid PyODT organic inhibitor. After careful analysis, we selected the Raman spectra that were found similar from all the investigated points for each class. These Raman data were used to create the training set; thus, for each class, 9 Raman spectra were employed, except the PyODT, for which the number of spectra was settled to 2 (firstly, because the PyODT organic substance is homogeneous, but especially, because this structure supports changes by dissolution in the 1.0 M HCl solution and after adsorption onto the electrode surface). Finally, the training dataset contained a total number of 47 Raman data. 

Further, the testing dataset was created in such a way to cover, for 5 of the 6 investigated classes (the PyODT class was not included in this group due to the considerations presented above), all the Raman profiles found on the C-steel surface. For the homogeneous surfaces, i.e., C-steel, the number of investigated points was lower (i.e., 3 Raman spectra). In contrast, for the other surfaces that possess different Raman features, more points were used in the study (i.e., 6 Raman spectra recorded from all-over treated C-steel surfaces). Finally, 27 points having similar or different Raman profiles with those from the C-steel surfaces found to be preponderant for a certain class were included in the testing group. 

In order to perform the ML study, we have used the Classification learner app implemented in MATLAB R2018b (MathWorks, Natick, MA, USA). During the study, for performing classification, several supervised learning models such as the decision trees, the discriminant analysis, the support vector machines (SVM), the nearest neighbor classifiers (KNN), and the ensemble classifiers have been employed.

## 3. Results and Discussion

### 3.1. Potentiodynamic Polarization

**Figure 2:**
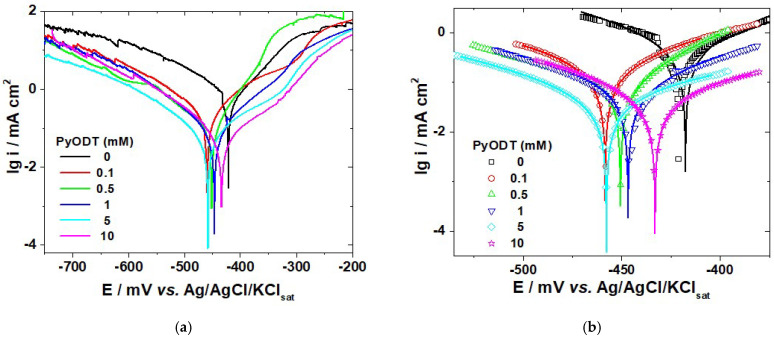
(**a**) Polarization curves of C-steel in 1.0 M HCl solution in the absence and the presence of different concentrations of PyODT.; (**b**) Comparison of the experimental and fitting data using a non-linear fitting procedure. Symbols = experimental data and lines = calculated data.

Potentiodynamic polarization curves of C-steel in 1 M HCl solution in the absence and presence of different concentrations of PyODT are shown in Figure 2. The electrochemical parameters, such as corrosion potential (*E_corr_*), anodic and cathodic activation coefficients (*b_a_* and *b_c_*), and corrosion current density (*j_corr_*) were estimated from the experimental data by applying a non-linear regression calculation near zero overall currents, based on the following Equation [35]:

*i* = *i_corr_*{exp[*b*_a_ (*E* − *E_corr_)*] − exp[*b*_c_ (*E* − *E_corr_*)]} (1)

The potential domain used in this calculation was limited to ±50 mV vs. *E_corr_* value to avoid the discrepancies which were sometimes noticed on the anodic or the cathodic branches of the polarization curves at high polarization. A similar approach was formerly applied in the case of C-steel [36] and several other metals corrosion [37]. The calculated electrochemical parameters are listed in Table 1. The values of the correlation factors ranging between 0.9963 and 0.9999 and the good overlapping between the calculated and the experimental polarization data (Figure 2b) confirm the accuracy of the fitting results.

The inhibition efficiency (*IE*) was estimated based on the *j_corr_* values, according to the following Equation:

(2) $$IE\;\left(\%\right)=\frac{j_{corr}^{0}-j_{corr}}{j_{corr}^{0}}\cdot 100$$

where $j_{corr}^{0}$ and $j_{corr}$ denote the corrosion current density values determined in the absence and presence of PyODT, respectively.

Inspection of Table 1 reveals a gradual decrease of the corrosion current density values in the presence of increasing PyODT concentrations compared to the blank sample. The lowest *j_corr_* value was obtained by the addition of 5 mM PyODT. At a higher concentration of inhibitor, an increase of the *j_corr_* value could be noticed in Table 1, although it remains much lower when compared to the uninhibited sample. At the same time, the corrosion potential shifts towards more negative values in the presence of the organic compound, but the displacement in the *E_corr_* values between the inhibited and uninhibited samples is less than ± 85 mV, suggesting that PyODT behaves as a mixed-type corrosion inhibitor. Furthermore, the changes in the cathodic and anodic activation coefficient values (Table 1) confirm that the PyODT influences the kinetics of the anodic and cathodic process. Since no definite trend was observed in the shift of *b_a_* and *b_c_* values in the presence of PyODT, it is reasonable to assert that the tested compound act as a mixed inhibitor in 1.0 M HCl corrosive environment. 

It should also be noted from Figure 2 that the anodic current densities measured at high anodic polarization in the presence of low concentrations of PyODT present greater values than those achieved in the blank electrolyte. A similar phenomenon was formerly reported for mild steel [38] and Q235 steel [2] corrosion and explained by the inhibitor desorption at large anodic polarization, which leads to a decrease of the surface coverage and an enhancement of the anodic dissolution reaction [38]. 

As shown in Table 1, the highest inhibiting efficiency of 91.2% was attained in the presence of 5 mM PyODT. The anticorrosive properties of PyODT could be related to its adsorption on the C-steel surface and the formation of a protective film, which might act as a barrier against the aggressive solution, and thus, reducing the metal corrosion rate. Further increase in the PyODT concentration to 10 mM leads to a slight decrease of its inhibiting efficiency.

It should also be mentioned that the anticorrosive performances of PyODT are similar to those reported by several selected 1,3,4-oxadiazole derivatives formerly investigated as corrosion inhibitors for steel materials in HCl solution (Table S1 from Supplementary Materials).

### 3.2. Electrochemical Impedance Spectroscopy

**Figure 3:**
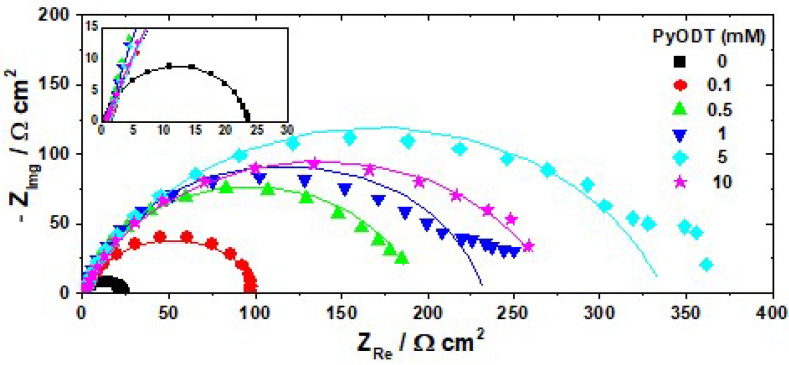
Nyquist diagrams recorded after 1 h immersion of C-steel surface in 1.0 M HCl solution in the absence and presence of different concentrations of PyODT. Insert: The impedance diagrams in an enlarged scale. Symbols–experimental impedance data; lines–calculated impedance.

Nyquist diagrams for C-steel obtained after 1 h stabilization period in 1.0 M HCl solution in the absence and in the presence of various concentrations of PyODT are given in Figure 3.

As illustrated in Figure 3, all impedance spectra are characterized by one capacitive loop with the center below the real impedance axis, which indicates the presence of a single charge-transfer process mainly controlling the corrosion of C-steel under the tested experimental conditions [39]. The depressed features of the semicircle in the Nyquist diagrams are characteristic for the solid electrodes exhibiting frequency dispersion of the interfacial impedance as a result of the chemical heterogeneity and roughness of the metallic surface, impurities or dislocations, grain boundaries, adsorption-desorption processes of inhibitor molecules, and the formation of porous layers [40].

The significant increase of the capacitive loops’ diameters noticed in the presence of PyODT as compared to its absence (Figure 3) confirms the anticorrosive proprieties of the investigated organic compound. Moreover, the similarity in the shapes of the impedance diagrams recorded in inhibited and uninhibited solutions suggests that the C-steel corrosion mechanism does not change after the PyODT addition. 

**Figure 4:**
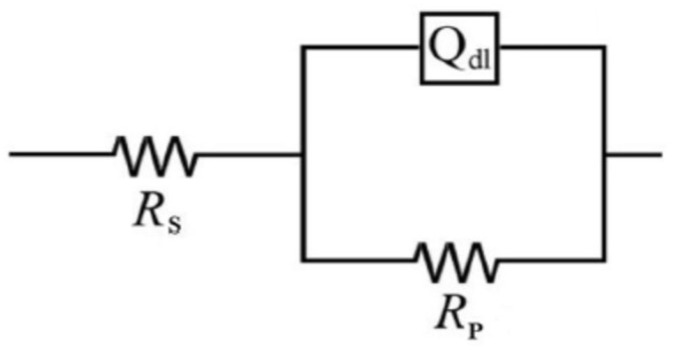
Equivalent electrical circuit model used for fitting the EIS experiment data.

The impedance spectra were further analyzed using the ZSimpwin software in terms of the equivalent electrical circuit depicted in Figure 4, in which *R_s_* stands for the solution resistance, *R_p_* represents the polarization resistance, and CPE is the constant phase element used instead of a pure capacitor (*C_dl_*) to obtain more accurate fitting results.

The impedance function of the CPE is mathematically expressed as follows:

Z_CPE_ = [Y(jω)*^n^*]^−1^ (3)

where ω = 2πf is the angular frequency, j = √(−1) is the imaginary unit, Y is the magnitude of the CPE, and *n* is a fitting parameter (0 ≤ *n* ≤1), which measure the element deviation from the ideal capacitive behavior (showing *n* = 1) [41].

The values of the double-layer capacitance (*C_dl_*) derived from the CPE parameters were calculated using Equation (4) [42]:

C_dl_ = (R^1−*n*^ Y)^1/*n*^ (4)

The electrochemical parameters calculated for C-steel corrosion in the absence and in the presence of PyODT are given in Table 2. The inhibition efficiency values (*IE*) estimated based on Equation (5) are also listed in Table 2.

(5) $$\mathrm{IE}\;\left(\%\right)=\frac{R_{p}-R_{p}^{0}}{R_{p}}\cdot 100$$

where $R_{p}$ and $R_{p}^{0}$ are the polarization resistances in solution with and without PyODT, respectively.

As readily seen in Figure 3, there is a satisfactory agreement between the experimental results (represented by symbols) and the calculated data (represented by lines), which confirms the validity of the proposed equivalent circuit model. Moreover, the small values of Chi-square (χ2) and the error percentages below 10% obtained for each component of the equivalent circuit demonstrate once more that the experimental EIS data adjusted well to the used fitting model. 

Table 2 shows that *R_p_* values become greater as PyODT concentration increases up to 5 mM. In comparison, no substantial improvement of the charge transfer process was observed at a higher concentration of inhibitor (10 mM). Since the corrosion rate is inversely proportional to the polarization resistance, the significant increase in the *R_p_* values clearly points out a marked inhibitive effect of PyODT on the C-steel corrosion. Likewise, the decrease in the *C_dl_* values noted in the presence of the organic inhibitor may be the consequence of a decrease in the local dielectric constant and/or an increase in the thickness of the electrical double layer at the metal-solution interface [43]. This behavior could result from the gradual replacement of water molecules and other ions originally present at C-steel/solution interface by the adsorbed PyODT molecules, which might form an inhibitor layer, leading to a smaller electrode area directly in contact with the aggressive acidic environment. The results are also consistent with the low values of *n* coefficient, indicating the heterogeneity of the electrochemical processes on the active sites. 

As shown in Table 2, the highest *IE* value reaches 93.1% in the presence of 5 mM PyODT, which was, therefore, deemed as the optimum concentration of inhibitor in the tested experimental conditions. In the investigated experimental conditions, the *IE* values were estimated from the potentiodynamic polarization measurements and in reasonable agreement with those achieved by EIS interpretation.

EIS measurements were further performed to investigate the effect of the immersion time on the corrosion behavior of C-steel in 1.0 M HCl solution without and the optimum concentration of PyODT. The impedance spectra were collected every hour in the first 12 h of immersion and then at 24 h.

**Figure 5:**
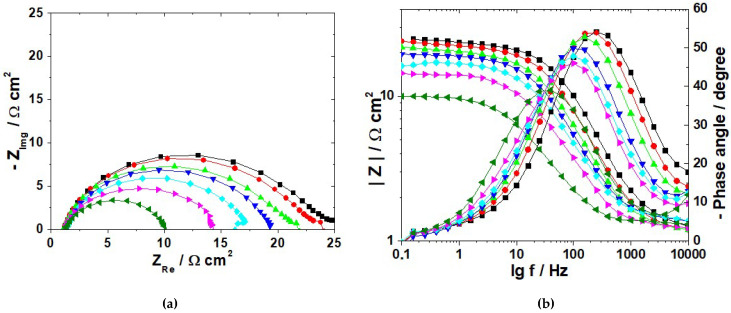

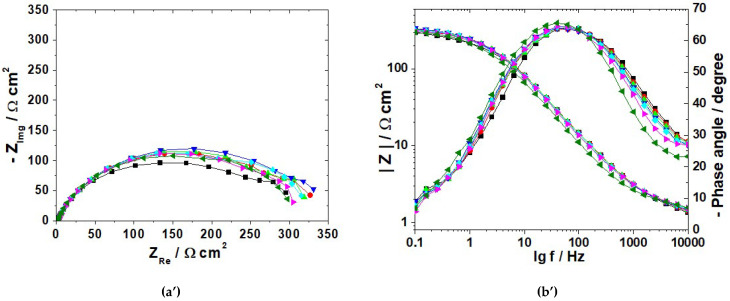
Nyquist and Bode plots of C-steel samples in 1.0 M HCl solution in the absence (**a,b**) and in the presence of 5 mM PyODT (**a’,b’**). The spectra were recorded at different immersion times in the test solutions (hours): (■) 2; (●) 4; (▲) 6; (▼) 8; (♦) 10; (►) 12; (◄) 24.

The time-evolution of the impedance in the blank and PyODT-containing solutions is reported in Figure 5. Several selected experimental data are presented as Nyquist and Bode plots, respectively.

The electrochemical behavior of the blank and inhibited specimens remained rather unchanged over the immersion period, revealing a single depressed capacitive loop in the entire frequency range corresponding to an activation-controlled corrosion process. 

The single peak observed in the Bode plots from Figure 5 confirms that the one-time constant equivalent circuit model (Figure 4) was properly used for the impedance fitting. Nevertheless, the changes in the magnitude of the impedance noticed at various exposure times endorses that the C-steel corrosion process is also time-dependent. 

As seen in Figure 5, a progressive decrease of the impedance modulus, |*Z*|, and a shift of the maximum phase angle towards lower values occurs at increasing exposure to the blank solution. This correlates with an important acceleration of the C-steel corrosion at prolonged immersion in 1.0 M HCl solution. In the presence of PyODT, regardless of its concentration, the |*Z*| values increase at all immersion times. At the same time, the corresponding maximum phase angles present higher values than the blank solution, advising for prolonged effective protection afforded by the tested organic inhibitor on the C-steel surface during the corrosion tests. 

**Figure 6:**
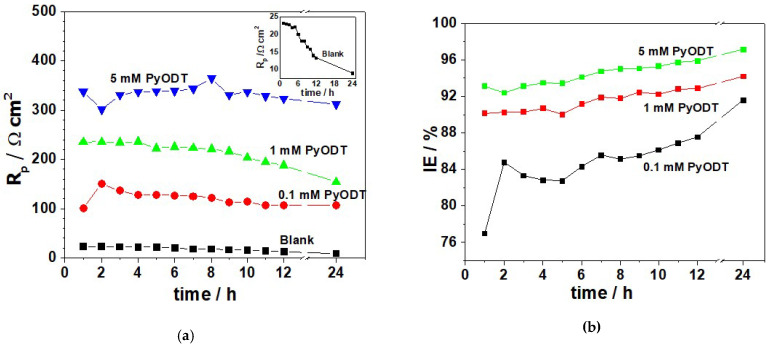
Variation of the polarization resistance (**a**) and inhibition efficiency (**b**) during the exposure of C-steel in 1.0 M HCl solution in the absence and the presence of selected concentrations of PyODT.

The calculated *R_p_* and *IE* values were plotted against the immersion time and presented in Figure 6. 

As illustrated in Figure 6, the *R_p_* values are higher in the presence of the PyODT than in its absence at all investigated immersion times and significantly increase with the inhibitor concentration. For a certain concentration of PyODT, the *R_p_* values do not change substantially within the exposure period, which suggests that the inhibitor adsorption on the C-steel surface was relatively fast and completed within the first hours of immersion. After that, the protective properties of the organic inhibitor remained rather constant by the end of the corrosion tests. The inhibition efficiencies were estimated using Equation (5) and found to reach the maxim values of about 95.9% and 97.1% in the presence of 5 mM PyODT, after 12 h and 24 h of immersion, respectively. However, it should be emphasized that the high values of *IE* were obtained merely based on the significant decrease of the *R_p_* values at prolonged exposure in the blank electrolyte.

### 3.3. Adsorption Isoterms

In corrosion studies, adsorption isotherms are commonly used to describe the interaction between the adsorbed inhibitor molecules and the metallic surface. It is known that the adsorption on the corroding surfaces never reaches the real equilibrium but tends to reach an adsorption steady state, which might become a quasi-equilibrium adsorption when the corrosion rate is sufficiently small [44]. In this case, it is reasonable to consider the quasi-equilibrium adsorption in a thermodynamic way using the appropriate equilibrium isotherms [44]. 

In an attempt to find the most suitable isotherm for the adsorption of PyODT on the C-steel surface, the results obtained from the electrochemical measurements were fitted into various isotherms, namely Langmuir, Temkin, and Frumkin. For this purpose, the values of surface coverage degree, θ, were calculated as a function of PyODT concentration, assuming that its inhibiting efficiency is mainly due to the blocking effect of the adsorbed organic molecules on the metal surface and hence *IE = 100 × θ* [45]. 

It was found that the values of the surface coverage (θ) (obtained from EIS measurements could be properly fitted to Langmuir adsorption isotherm, which is expressed by Equation (6):

(6) $$\frac{c_{Inh}}{\theta }=\frac{1}{K}+c_{Inh}$$

where *c_Inh_* is the inhibitor concentration (M), and K represents the equilibrium adsorption constant.

**Figure 7:**
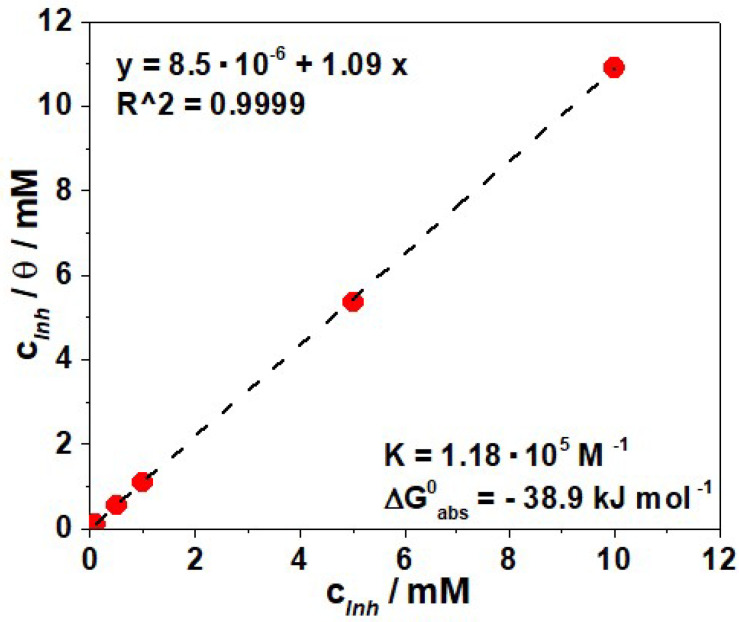
Langmuir adsorption isotherm for C-steel in 1.0 M HCl solution containing PyODT.

As shown in Figure 7, the plot of $\frac{c_{Inh}}{\theta }$ versus c*_Inh_* yields to a straight line with nearly unit slope and a correlation coefficient close to 1.00, which confirms that the experimental data corresponding to PyODT adsorption on C-steel surface are well described by the Langmuir isotherm approach. This model assumes that the adsorbing species form only a monolayer coverage on the metal surface and no interactions between the adsorbed species on the electrode surface occur [14]. However, in real conditions, some interactions between the adsorbing species occur on the metal surface since the organic molecules usually possess polar groups that could interplay through common repulsion or attraction forces, causing deviations of the slope of the Langmuir plot from unity [46], as noticed in Figure 6. 

The value of the equilibrium constant (K = 1.18 × 10^5^ M^−1^) obtained from the Langmuir plot is high and implies strong interactions between the organic inhibitor molecules and the metal surface. The standard free energy of adsorption $\Delta G_{ads}^{0}$ was further calculated based on the *K* value, according to Equation (7):

(7) $$K=\frac{1}{55.5}\exp\left(\frac{-\Delta G_{ads}^{0}}{RT}\right)$$

where 55.5 represents the molar concentration of water in solution (mol L^−1^), *R* is the gas constant, and *T* is the absolute temperature (298 K).

Generally, the value of $\Delta G_{ads}^{0}$ are used to ascertain the nature of the adsorption process; if $\Delta G_{ads}^{0}$ is less negative than −20 kJ mol^−1^, the interaction of the inhibitor molecules with the metal surface is mainly based on the physical adsorption, while if $\Delta G_{ads}^{0}$ is more negative than −40 kJ mol^−1^, the charge sharing from the inhibitor to the metal could cause chemisorption. When the value of $\Delta G_{ads}^{0}$ is between −20 and −40 kJ mol^−1^, both, physical and chemical adsorptive interactions might be presumed.

In this work, the calculated value of $\Delta G_{ads}^{0}$ was −38.9 kJ mol^−1^. This large negative value of $\Delta G_{ads}^{0}$ reveals the spontaneity of the adsorption process and the stability of the adsorbed inhibitor layer on the metal surface. Moreover, based on the value of $\Delta G_{ads}^{0}\;$, two modes of interaction, i.e., physisorption and chemisorption, can be assumed to explain the adsorption behavior of PyODT on the C-steel surface.

### 3.4. Surface Investigations

#### 3.4.1. SEM and EDX Analysis

**Figure 8:**
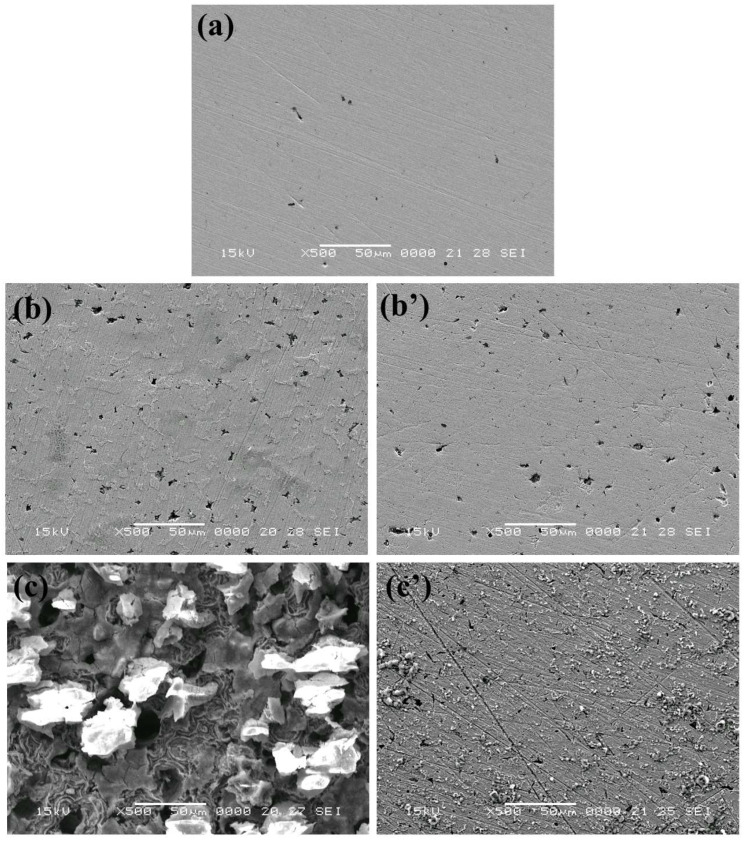
SEM images of C-steel surface before (**a**) and after 1 h (**b**,**b’**) and 12 h (**c**,**c’**) of immersion in 1.0 M HCl solution in the absence (**b**,**c**) and in the presence of 5 mM PyODT (**b’**,**c’**).

**Figure 9:**
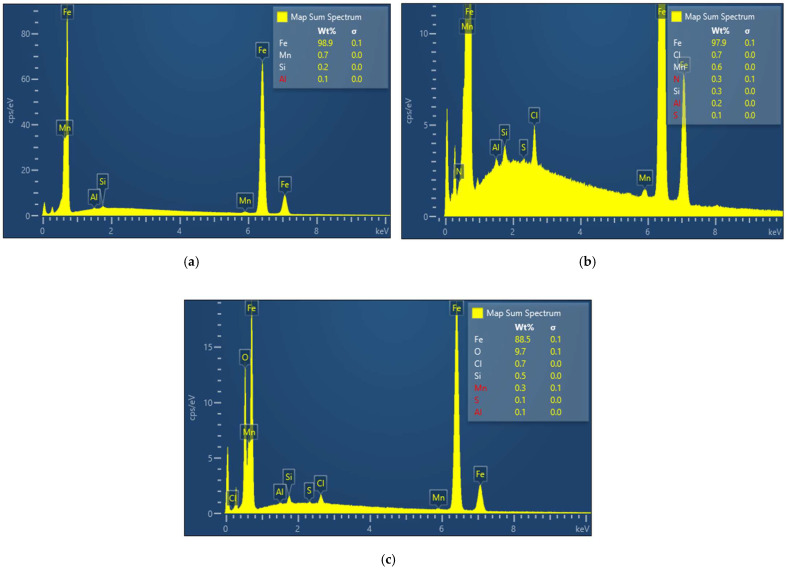
EDX spectra of C-steel surface before (**a**) and after 1 h (**b**) and 12 h (**c**) immersion in 1.0 M HCl solution containing 5 mM PyODT.

SEM observations (Figure 8) and EDX analyses (Figure 9 and Table 3) were carried out on the C-steel surface before and after 1 h and 12 h immersion in 1.0 M HCl solution without and with the addition of the ‘optimum’ concentration of PyODT.

As expected, before immersion in the corrosive solution, the C-steel specimen presents a smooth surface with slightly visible abrading scratches. However, some defects, such as voids, could be noticed in Figure 8a.

After 1 h exposure to 1.0 M HCl solution, the surface of the C-steel appears covered with corrosion products, while several pits are clearly visible in Figure 8b. In contrast, in the presence of PyODT, the metallic surface seems to be not affected by corrosion (Figure 8c). The results are supported by the EDX analysis revealing the presence of the nitrogen and sulfur peaks, which demonstrate the presence of PyODT in the surface layer (Figure 9b and Table 3). Furthermore, the absence of oxygen in the surface film suggests that the adsorption of PyODT is fast and prevents the formation of corrosion products (oxides). Therefore, it can be concluded that a barrier film resulting from the adsorption of PyODT was formed on the C-steel surface, in agreement with the electrochemical results.

As expected, the surface of the C-steel appears significantly damaged after 12 h of exposure to the uninhibited solution, exposing several deep and large cavities, along with a thick and uneven layer of corrosion products (Figure 8c). EDX investigations also reveal large amounts of oxygen and chloride on the metal surface, confirming the significant extent of the corrosive attack (Table 3). Moreover, the sharp decrease in the Fe content attests to the formation of a thick layer of corrosion products at 12 h exposure time.

The surface of the PyODT-inhibited metallic specimen is significantly improved, showing only traces of corrosion products, while the polishing scratch-lines are still visible after 12 h exposure (Figure 8c’). In addition, the important reduction in the oxygen and chloride content noticed in Figure 9c and Table 3 demonstrates the ability of the adsorbed PyODT molecules to suppress the C-steel corrosion at extended exposure. The absence of some inhibitor elements on the surface might indicate the formation of a very thin layer on the surface, reducing then the resolution on analyzing these elements.

#### 3.4.2. Raman Spectroscopy and Machine Learning Investigations

##### Raman Spectroscopic Evaluation of the Electrodes’ Surface

**Figure 10:**
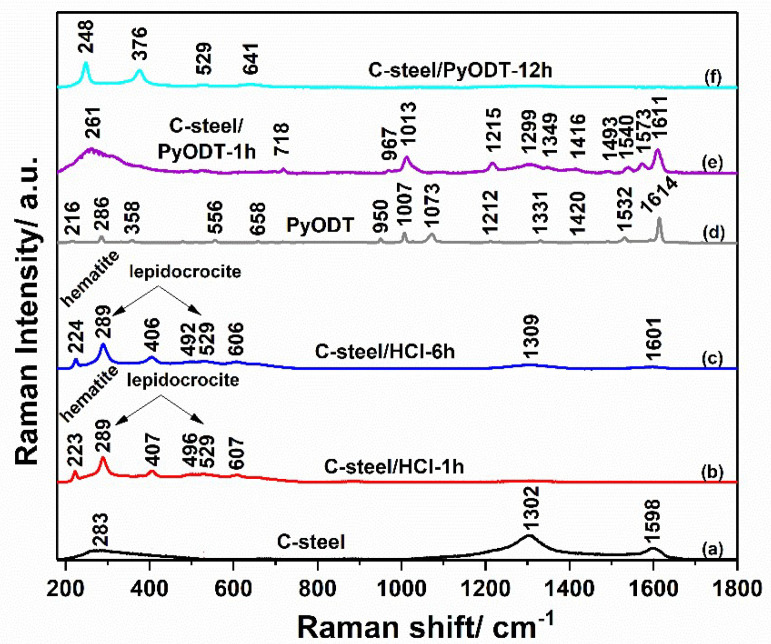
Raman spectra of the C-steel surface before (**a**) and after exposure to 1.0 M HCl solution for 1 h (**b**) and 6 h (**c**), of the PyODT powder (**d**), and the C-steel surface after 1 h (**e**) and 12 h (**f**) exposure to 5 mM PyODT-containing solution.

The C-steel surface was further investigated by Raman spectroscopy in order to follow the transformation occurring on the metal during its exposure to 1.0 M HCl solution, in the absence and the presence of the organic inhibitor. Figure 10 shows the Raman spectra of the C-steel surface before (a) and after different immersion times in the uninhibited (b, c) and 5 mM PyODT-containing solutions (e, f).

**Figure 11:**
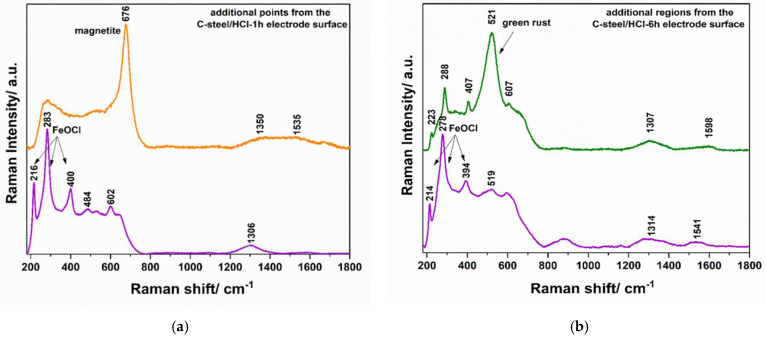
Raman spectra recorded for various additional regions of the C-steel after 1 h (**a**) and 6 h (**b**) exposure to 1 M HCl solution.

After a brief evaluation of the Raman spectra, it appeared clear that various stages could be identified onto the electrodes’ surface. The Raman spectrum of the C-steel electrode immersed for 1 h in 1.0 M HCl solution, Figure 10b indicates the spectral features of the hematite and lepidocrocite products. Thus, the peaks from 223, 289, 407, 496, and 607 cm^−1^ could be associated with hematite (α-Fe_2_O_3_). In comparison, the bands from about 289 (superposition of peaks) and 529 cm^−1^ suggest the presence of the lepidocrocite (γ-FeOOH) compound on the metal surface in the investigated point (the major bands of the iron oxyhydroxide lepidocrocite appears at 284 and 524 cm^−1^) [47]. In addition to the common hematite and lepidocrocite mixture preponderantly identified inside the corrosion area formed on the C-steel surface after 1 h exposure to 1.0 M HCl solution, a more detailed spectroscopic investigation of the electrode surface revealed the existence of at least another two types of iron oxides (Figure 11a); however, these compounds appeared to a lesser extent in the investigated regions. According to the literature data [47,48,49], a mixture of the iron oxychloride (FeOCl), as a major compound, and hematite, as a minor product, could also be found in some regions of the electrode surface. At the same time, rarely, in others, the magnetite (Fe_3_O_4_) was clearly identified on the electrode surface. The presence of the magnetite on the C-steel is not an uncommon phenomenon if the solutions are oxygen-deficient systems and contain not enough oxygen to conduct to the common rust (Fe_2_O_3_).

By increasing the exposure time of the C-steel surface to the blank solution up to 6 h, the corrosion regions became predominant on the metal. Figure 10c indicates the presence of hematite as the main product, and lepidocrocite, as a minor component, on the electrode surface [49]. The iron oxychloride (FeOCl) and the green rust were also identified on the C-steel surface after 6 h of immersion in 1.0 M HCl solution, as shown in Figure 11b [47,50]. This rapid extension of the corrosion process in the presence of chloride ions is a well-known phenomenon, being supported by the fact that chloride is a relatively small electronegative ion that can diffuse easily through the pores and accelerates the hydrolysis of Fe^2+^ ions [51]. 

The Raman profiles of the C-steel surface recorded after 1 h and 12 h of exposure to 5 mM PyODT-containing solution are illustrated in Figure 10e,f. The increase of the exposure time to the inhibitor-containing solution to 12 h instead of 6 h (the time selected for the examination of the corrosion extent of C-steel surface exposed to the blank solution) was imposed by the necessity to have a sufficiently long period for the proper evaluation of the surface film stability towards the chloride ions attack. The obtained results indicate the adsorption of the PyODT molecules on the C-steel surface in the first hour of immersion, followed by a re-organization of the organic molecules as the exposure time increases to 12 h. Thus, as it can be seen in Figure 10, significant differences are observed between the Raman spectra of solid PyODT (Figure 10d) and the species found on the metal surface after 1 h of immersion in PyODT-containing solution (Figure 10e). The bands from 718, 967, 1013, 1215, 1349, 1416, 1493, 1540, 1573, and 1611 cm^−1^ can be attributed to the vibrational modes of the adsorbed-PyODT molecules, showing that the PyODT molecule did not preserve the structure of the PyODT powder. A similar situation has been found after the adsorption of PyODT on the copper electrode. Still, a comparison between the Raman profiles of the adsorbed-PyODT species on the copper and C-steel electrodes suggests the existence of two different structures on the surface of the two metallic electrodes [34]. These differences might result from the protonation process that occurs in the 1.0 M HCl electrolyte used for the current study.

Although, for the C-steel/PyODT-12h electrode, the molecular structure of the film formed on its surface was not elucidated yet, some data, such as the two peaks from 248 and 376 cm^−1^, could indicate the presence of the Fe-N vibration modes, similar to that found in the Fe-imidazole interactions [52] and to the asymmetric Fe-S stretching vibrations [53], respectively. Due to the molecular adsorption and structural organization of the PyODT molecules, it was possible to obtain a more organized film that is able to limit to a greater extent the Cl^−^ ions attack, as well as the damage of the PyODT film, hindering the C-steel corrosion, in agreement with the results of the SEM-EDX investigations.

**Figure 12:**
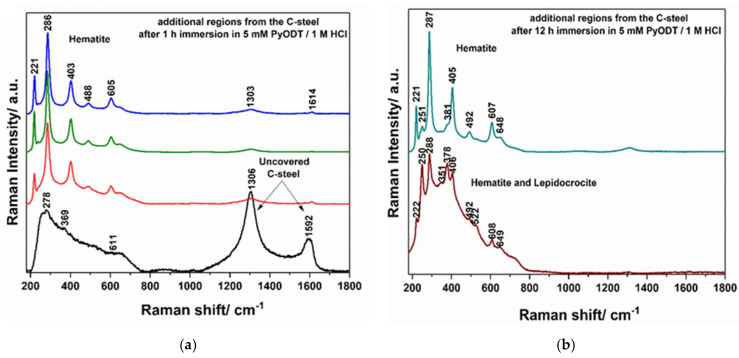
Raman spectra recorded on various additional regions of the C-steel after 1 h (**a**) and 12 h (**b**) of exposure to 5 mM PyODT-containing solution.

Moreover, a detailed spectroscopic evaluation of the C-steel electrode surface after its immersion in a 5 mM PyODT-containing solution showed some important differences in the corrosion sites and the exposure time. Thus, although the Raman measurements carried out after 1 h immersion in the corrosive solution containing 5 mM PyODT indicate the presence of the inhibitor film on the C-steel surface, some uncovered areas together with several corrosion sites could be noticed in Figure 12a. Extending the immersion time to 12 h leads to a more compact PyODT film and the lack of C-steel uncovered regions. However, the presence of several corrosion sites can be detected even here, in accordance with the SEM-EDX results. These corrosion sites are mainly composed of hematite and mixtures of hematite and lepidocrocite compounds, as illustrated in Figure 12b. These results explain the inhibiting efficiency values lower than 100% obtained by the electrochemical investigation.

##### Electrode Surface Evaluation Using Machine Learning Approach

Since the PyODT film does not entirely hinder the chloride ions attacks, the rapid identification of the corrosion sites on the C-steel surface could be useful for the correct evaluation of the metal degradation state. Considering the results obtained by the Raman spectroscopic technique, which allows the discrimination between the PyODT-film and corrosion products (Figure 10, Figure 11 and Figure 12), we further initiated a study focused on the use of Raman spectra in combination with Machine Learning (ML) models for the C-steel surface state evaluation. 

For this purpose, we tested the possibility of obtaining a good identification of the investigated areas through their Raman spectra and the ML algorithms implemented in Matlab R2018b. Thus, we selected six classes of interest, namely untreated C-steel (CS), C-steel after 1 h and 6 h of immersion in 1.0 M HCl (CS-HCl-1h and CS-HCl-6h), C-steel after 1 h and 12 h of immersion in 1.0 M HCl solution containing 5 mM PyODT (CS-PyODT-1h and CS-PyODT-12h) and the solid PyODT. For each class, we recorded Raman spectra at different points, and further, after the spectra pre-treatment, we identified those spectra that are specific to it. These spectra have been used to create the training dataset; finally, the training group contains 47 Raman spectra (see Experimental section). 

For the testing dataset preparation, several spectra possessing different profiles were added to the main Raman profiles (which were similar to those identified and used for the training group creation). Thus, a total number of 27 Raman spectra associated with 5 of the 6 investigated classes were evaluated to establish the predicted model’s ability to correctly identify the type of the investigated areas (see Experimental details). Table 4 contains the number of the variables used in the ML investigation; these data indicate the total training or testing numbers of the Raman spectra employed for each class.

**Figure 13:**
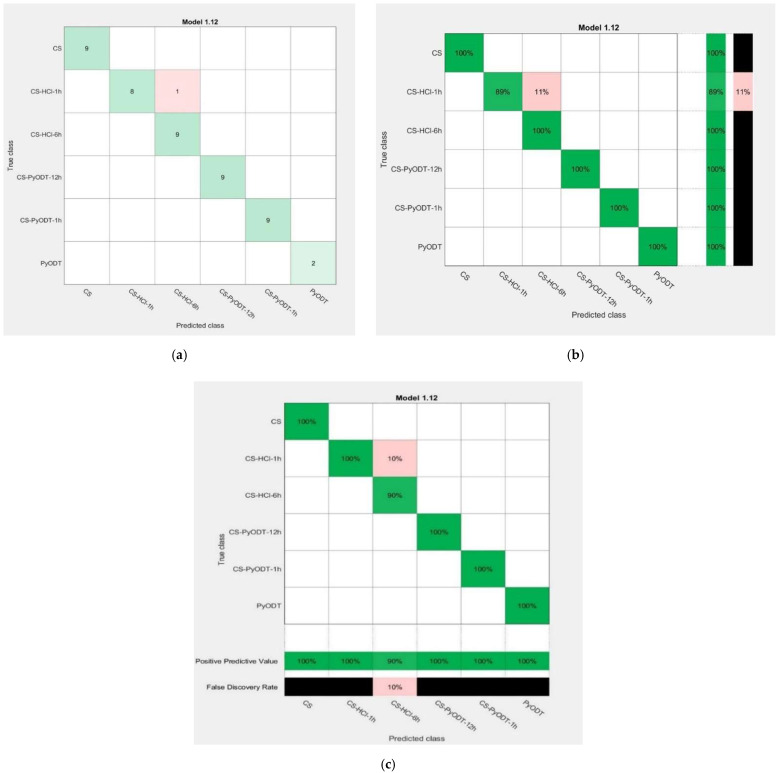
Confusion matrix obtained for the five types of surfaces found onto the C-steel electrode during the study and solid PyODT-corrosion inhibitor; classification indicated as the number of observations (**a**), true positive vs. false-negative rates (**b**), or positive predicted value vs. false discovery rate (**c**).

Figure 13 shows the confusion matrix for the best prediction model, in which accuracy of 97.9% was obtained using a 6-fold cross-validation procedure and the KNN fine method (the number of neighbors is 1, distance metric–Euclidean, distance weight–equal and standardized data–true). These results are acquired by training the 47 Raman spectra selected for the training group (Table 4).

This model allowed us to discriminate between the five classes identified in significant proportions onto the electrode surface after the various treatments applied and the organic inhibitor solid structure. The results are indicated in Figure 13a as the number of the used observations; the green line evidences the right classification of samples, while the red color is used for the wrong sample attribution. According to these results, the best classification model accuracy of 97.9% was obtained after the wrong classification of a sample from the CS-HCl-1h class which was assigned to the CS-HCl-6h group. This conducts to an 89% true positive rate for the CS-HCl-1h class, which shows the probability that a point of this class to be correctly identified as belonging to the proper category (Figure 13b). However, we assumed that this rate and the false discovery rate value of 10% (Figure 13c) are rather limitations due to the specificity of the electrode surface after the aggressive treatment in 1.0 M HCl solution. It appears that, on the C-steel surface, there are regions with severe corrosion after 1 h exposure to the blank solution, similar to that obtained after 6 h of immersion.

This trained model was exported into the workspace in order to make predictions using the new data from the testing dataset (Table 4). The obtained results are presented in Table 5 and indicate the correct assignment for all the CS (C-steel) points; the electrode surface homogeneity was already observed during its investigation. For the other classes, the results show at least a 50% good prediction percentage, except the **CS-PyODT-1h group** for which only 1 point of the six ones used in the testing set is correctly indicated as belonging to its class. Thus, in Table 5, the ‘**true class**’ indicates that the investigated point belongs to a certain class (i.e., CS-HCl-1h means that the spectrum of the point was registered by analyzing the CS-HCl-1h electrode surface), while the ‘**predicted class**’ specifies the group category after predicting on the new Raman dataset. Moreover, in Table 5, the good predicted results are bolded, while those predictions that do not match the investigated class are indicated in italics.

**Figure 14:**
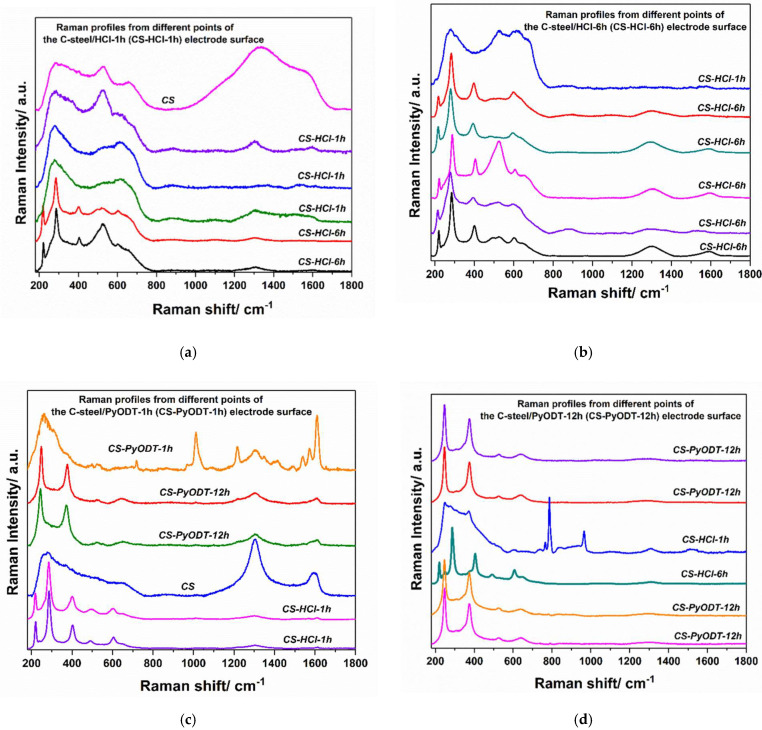
Raman spectra of different points from the C-steel surface after various treatments: 1 h immersion in 1.0 M HCl (**a**); 6 h immersion in 1.0 M HCl (**b**); 1 h immersion in 5 mM PyODT-containing solution (**c**); 12 h immersion in 5 mM PyODT-containing solution (**d**). The *italic* notation from each spectrum indicates the prediction obtained after evaluating the best-performing model (KNN) on the testing dataset.

However, to better understand the results, the Raman profiles of the investigated samples are illustrated in Figure 14 in accord with their order in the testing dataset and Table 5 occurrence (for simplicity, the CS class was excluded from analysis). The italics labels from each spectrum indicate the prediction obtained after testing these new Raman data (testing dataset), Figure 14. 

After a detailed analysis of the prediction acquired for the testing set samples and the Raman profiles indicated in Figure 14, for the four classes, CS-HCl-1h, CS-HCl-6h, CS-PyODT-1h, CS-PyODT-12h, it is clear that the data that appeared as being a wrong assignment in Table 5, in effect, represent a correct identification of the surface compounds, in accordance with their Raman profile. For example, in the case of the CS-PyODT-1h, the prediction model suggests the existence of different compounds on the CS-PyODT-1h surface. Thus, after the Raman spectra analysis of all the points used in the testing dataset, we observed that one point possesses similar features with the CS-PyODT-1h, 2 points show the profile of the CS-PyODT-12h surface, one point has the main bands of the CS. In contrast, the other two points contain the main peaks of the hematite species that can be found either in the CS-HCl-1h or CS-HCl-6h samples, Figure 14c. In this situation, we assume that the prediction results obtained on the new data are in accord with the real profile of the analyzed points and allow us to conclude that by using Raman spectroscopic investigations in combination with ML algorithms, a rapid and reliable detection of the corrosion points can be easily achieved.

## 4. Conclusions

The inhibiting performances of 5-(4-pyridyl)-1,3,4-oxadiazole-2-thiol (PyODT) against C-steel corrosion in 1.0 M HCl solution were investigated by electrochemical measurements, surface analysis, and machine learning algorithms. 

The electrochemical results demonstrate that PyODT can effectively inhibit the C-steel corrosion process, and its maximum protection efficiency of 93.1% was attained after 1 h exposure in the corrosive solution containing 5 mM PyODT. An enhancement of the PyODT inhibiting efficiency to about 97% was observed at 24 h exposure. The potentiodynamic polarization measurements revealed that PyODT is mainly a mixed-type corrosion inhibitor in a 1.0 M HCl solution.

The surface characterization by SEM-EDX and Raman spectroscopy confirms the inhibitor adsorption and the formation of a protective film on the metal surface. The adsorption of PyODT obeys the Langmuir isotherm and the estimated value of $\Delta G_{ads}^{0}$ further suggests the combination of physisorption and chemisorption processes.

Furthermore, an approach based on Raman spectra in combination with machine learning algorithms was applied to evaluate and follow the surface degradation of the C-steel exposed to a highly aggressive environment and various experimental conditions. The KNN algorithm allowed a reliable recognition of the Raman spectra corrosion sites (accuracy 97.9% and correct identification of the surface compounds from the testing group), demonstrating that this approach might offer a facile and rapid tool for the early identification of the incipient corrosion damages of the C-steel surfaces.

## Figures and Tables

**Table 1:** Electrochemical parameters of C-steel in 1 M HCl solution, in the absence and the presence of various concentrations of PyODT, determined from the polarization curves.

PyODT (mM)	*E_corr_* (mV vs. Ag/AgCl/KCl_sat_)	*j*_corr_ (μA cm^−2^)	*b_a_* (V^−1^)	*-b_c_* (V^−1^)	IE (%)
0	−417.1 ± 0.18	856.6 ± 0.27	25.58 ± 0.62	21.39 ± 0.50	-
0.1	−458.5 ± 0.01	300.3 ± 1.56	22.05 ± 0.08	19.24 ± 0.10	64.9
0.5	−450.3 ± 0.03	137.7 ± 0.07	39.84 ± 0.09	15.53± 0.12	83.9
1.0	−446.5 ± 0.01	111.4 ± 4.00	26.22 ± 0.66	20.87 ± 0.70	86.6
5	−458.1 ± 0.34	75.8 ± 0.79	19.80 ± 0.14	19.50 ± 0.17	91.2
10	−432.8 ± 0.02	83.9 ± 0.04	15.36± 0.08	22.03 ± 0.06	90.2

**Table 2:** Impedance parameters for C-carbon steel corrosion in the absence and the presence of PyODT at different concentrations.

PyODT (mM)	*R_p_* (Ω cm^2^)	*Q_dl_* (μF s^n−1^ cm^−2^)	*n_dl_*	*C_dl_* (μFcm^−2^)	IE (%)
0	23.2	1090.0	0.832	519.0	-
0.1	100.8	387.4	0.828	197.7	76.9
0.5	192.5	107.8	0.860	57.4	87.9
1.0	235.4	92.1	0.851	47.1	90.1
5	337.5	138.2	0.709	39.3	93.1
10	273.8	600.0	0.775	354.4	91.5

**Table 3:** EDX analysis of C-steel surface after different immersion times in 1.0 M HCl solution without and with 5 mM PyODT, as determined from EDX analysis.

Corrosive Solution	Element (wt.%)							
	Fe	O	Cl	Mn	Si	N	S	Al ^1^
1 h immersion								
1.0 M HCl	92.7	1.2	5.1	0.5	0.2	-	-	0.2
1.0 M HCl + 5 mM PyDOT	97.9	-	0.7	0.6	0.3	0.3	0.1	0.2
12 h immersion								
1.0 M HCl	67.3	24.9	7.4	0.3	0.1	-	-	-
1.0 M HCl + 5 mM PyDOT	88.5	9.7	0.7	0.3	0.5	-	0.1	0.1

^1^ Traces of Al from the grinding procedure.

**Table 4:** Machine learning data, number of Raman spectra–total/training/testing used for each class.

Class	C-steel (CS)	CS-HCl-1h	CS-HCl-6h	CS-PyODT-1h	CS-PyODT-12h	PyODT
Number of variables	12/9/3	15/9/6	15/9/6	15/9/6	15/9/6	2/2/-

**Table 5:** C-steel surface analysis: results obtained to evaluate the best-performing model (KNN) on the testing dataset.

Predicted Class	True Class	Predicted Class	True Class
**CS**	**CS**	**CS-HCl-6h**	**CS-HCl-6h**
**CS**	**CS**	**CS-PyODT-1h**	**CS-PyODT-1h**
**CS**	**CS**	*CS-PyODT-12h*	*CS-PyODT-1h*
*CS-HCl-6h*	*CS-HCl-1h*	*CS-PyODT-12h*	*CS-PyODT-1h*
*CS-HCl-6h*	*CS-HCl-1h*	*CS*	*CS-PyODT-1h*
**CS-HCl-1h**	**CS-HCl-1h**	*CS-HCl-1h*	*CS-PyODT-1h*
**CS-HCl-1h**	**CS-HCl-1h**	*CS-HCl-1h*	*CS-PyODT-1h*
**CS-HCl-1h**	**CS-HCl-1h**	**CS-PyODT-12h**	**CS-PyODT-12h**
*CS*	*CS-HCl-1h*	**CS-PyODT-12h**	**CS-PyODT-12h**
*CS-HCl-1h*	*CS-HCl-6h*	*CS-HCl-1h*	*CS-PyODT-12h*
**CS-HCl-6h**	**CS-HCl-6h**	*CS-HCl-6h*	*CS-PyODT-12h*
**CS-HCl-6h**	**CS-HCl-6h**	**CS-PyODT-12h**	**CS-PyODT-12h**
**CS-HCl-6h**	**CS-HCl-6h**	**CS-PyODT-12h**	**CS-PyODT-12h**
**CS-HCl-6h**	**CS-HCl-6h**		

## Data Availability

Data sharing is not applicable for this article.

## Supplementary Materials

The following supporting information can be downloaded at: https://www.mdpi.com/article/10.3390/ma15062224/s1, Table S1. Inhibition performances for several studied 1,3,4-oxadiazole derivatives on mild steel corrosion in HCl solution.
