# Supplementary material for: Combined Electrochemical, Raman Analysis and Machine Learning Assessments of the Inhibitive Properties of an 1,3,4-Oxadiazole-2-Thiol Derivative against Carbon Steel Corrosion in HCl Solution
Source: Materials (Basel). 2022 Mar 17;15(6):2224. doi: 10.3390/ma15062224 (PMC8950990; doi:10.3390/ma15062224)
Supplement: Supplementary file 1 [file materials-15-02224-s001.zip › materials-1559394-supplementary.pdf]

Supplementary Material

Table S1. Inhibition performances for several studied 1,3,4-oxadiazole derivatives on mild steel corrosion in HCl solution.

Inhibitor	Electrolyte	Inhibitor Concentration Range	Investigation Techniques	Inhibitor Performances	Ref.
2,5-bis(2-aminophenyl)-1,3,4-oxadiazole (2-APOX)	1.0 M HCl	20-100 mg/L	Gravimetric measurements<br>EIS, Polarization curves	IE * = 81.4 – 92.4 %<br><br>Mixed-type inhibitor<br>Adsorption of 2-APOX obeys Langmuir isotherm<br>Temperature-independent inhibition efficiency<br>2-APOX is a noncytotoxic substance	[21]
2,5-bis(2 pyridyl)-1,3,4-oxadiazoles (2-POX)<br><br>2,5-bis(3 pyridyl)-1,3,4-oxadiazoles (3-POX)<br><br>2,5-bis(4 pyridyl)-1,3,4-oxadiazoles (4-POX)	1.0 M HCl	0.08-0.32 mM	Gravimetric measurements<br>EIS, Polarization curves<br>XPS	IE = 84.3 – 91.5 % (2-POX)<br>IE =82.6 – 90.1 % (3-POX)<br>IE = 81.4 – 86.9 % (4-POX)<br><br>Ability of the molecules to chemisorb on Fe surface depends on the position of N atom on the pyridyl substituent.<br>2-POX is the best inhibitor and acts as a mixed-type inhibitor.	[22]
2,5-bis(2-pyridyl)-1,3,4-oxadiazole (2-POX)<br><br>2,5-bis(2-hydroxyphenyl)-1,3,4-oxadiazole (2-HPOX)	1.0 M HCl	20-80 ppm	Gravimetric measurements<br>EIS, Polarization curves	IE = 84.3 – 91.5 % (2-POX)<br>IE= 85.9 – 94.7 % (2-HPOX)<br><br>2-HPOX molecule is stabilized by intramolecular hydrogen bond.<br>The better inhibiting properties of 2-HPOX are due to the presence of the electron releasing -OH group in the ortho position of the aromatic ring.	[23]
2,5-bis(4-nitrophenyl)-1,3,4-oxadiazole (PNOX)<br><br>2,5-bis(4-aminophenyl)-1,3,4-oxadiazole (PAOX)	1.0 M HCl	0.1-1 mM	Gravimetric measurements<br>EIS, Polarization curves<br>Quantum chemical calculations	IE = (-21.8) – (-9) % (PNOX)<br>IE = 85.3 – 94.1 % (PAOX)<br><br>PAOX acts as mixed-type inhibitor.	[24]

				PAOX adsorption obeys Langmuir isotherm with a physisorption process.<br>PNOX accelerates the corrosion process.	
2,5-bis(2-methoxyphenyl)-1,3,4-oxadiazoles (2-MOX)<br><br>2,5-bis(3-methoxyphenyl)-1,3,4-oxadiazoles (3-MOX)<br><br>2,5-bis(4-methoxyphenyl)-1,3,4-oxadiazoles (4-MOX)	1.0 M HCl	20-60 mg/L	Gravimetric measurements<br>EIS, Polarization curves<br>Quantum chemical calculations	IE ** = 97.9 – 99.8 % (2-MOX)<br>IE ** = 95.0– 97.6 % (3-MOX)<br>IE ** = 94.5 – 97.9 % (4-MOX)<br><br>The improved inhibiting effect of 2-MOX is due to the ortho position of the substituent and to the cationic form of this molecule highly stabilised by hydrogen bonding.<br>Adsorption of 2-MOX follows Langmuir isotherm.	[25]
2-cinnamyl-5-mercapto-1,3,4-oxadiazole (CMO)<br><br>2-hydroxyphenyl-5-mercapto-1,3,4-oxadiazole (HMO)<br><br>2-phenyl-5-mercapto-1,3,4-oxadiazole (PMO)	0.5 M HCl	500 ppm (optimum concentration)	Gravimetric measurements<br>Polarization curves	IE = 82.8 % (CMO)<br>IE = 71.4 % (HMO)<br>IE = 77.1% (PMO)<br><br>Cathodic inhibitors<br>Adsorption obeys Temkin's isotherm.<br>The highest inhibiting efficiency of CMO is due to the additional π-bond between the C atoms in conjugation with the aromatic ring, offering extensively delocalized π-electrons.<br>The orientation of the substituted groups in HMP prevent their flat positioning on metal surface, causing reduced adsorption.	[26]
5-(2-hydroxyphenyl)-1,3,4-oxadiazole-2-thiol	1.0 M HCl	0.1 - 0.5 mM	Gravimetric measurements<br>EIS, Polarization curves<br>XPS	IE = 62.19 – 90.79 %<br><br>Mixed-type inhibitor<br>Physisorption mechanism<br>Adsorption follows Langmuir isotherm.	[27]
2-methyl-2-{4-[5-(6-methyl pyridin-2-yl)-[1,3,4]oxadiazol-2-yl]-phenyl}-propionitrile (6 MMPO)<br><br>2-methyl-6-(5-pyridin-4-yl)-[1,3,4]oxadiazol-2-yl]-pyridine (5-MPOP)	0.5 M HCl		Gravimetric measurements<br>EIS, Polarization curves<br>SEM<br>Quantum chemical calculation	IE = 69.78 – 89.67 % (MMPO)<br>IE = 76.0 – 86.9% (5-MPOP)<br>IE = 53.0 – 84.6 % (4-BPOMP)<br><br>6-MMOPP > 4-BPOMP > 5-MPOP<br><br>Mixed-type inhibitors.<br>IE decreases with the temperature.	[28]

2-[5-(4-bromo-phenyl)-[1,3,4]oxadiazol-2-yl]-6-methyl-pyridine (4-BPOMP)				Physisorption mechanism	
5-((2-methyl-1Hbenzo[d]imidazol-1-yl) methyl)-1, 3, 4 oxadiazole-2-thiol	0.5 M HCl<br>1.0 M HCl<br>and<br>1.5 M HCl	200 ppm (optimum concentration)	Gravimetric measurements<br>EIS, Polarization curves<br>SEM<br>Quantum chemical calculations<br>Molecular dynamics simulations	IE = 92.2 % (in 0.5 M HCl)<br>IE = 85.30 % (in 1.0 M HCl)<br>IE = 83.50 % (in 1.5 M HCl)<br><br>Mixed-type inhibitor.<br>Adsorption follows Langmuir isotherm with a comprehensive physical and chemical mechanism.<br>Thione form is the most reactive tautomer, presenting a higher tendency to get adsorbed on metal as compared to thiol form, both in the neutral and protonated conditions.	[29]

* The inhibition efficiency (IE) values were determined at 30 ^{0}C, from the potentiodynamic polarisation measurements, except if other method is specified. ** IE values were determined at 30 ^{0}C, from the weight loss measurements.
